# MERS-CoV Infection of Alpaca in a Region Where MERS-CoV is Endemic

**DOI:** 10.3201/eid2206.152113

**Published:** 2016-06

**Authors:** Chantal B.E.M. Reusken, Chrispijn Schilp, V. Stalin Raj, Erwin De Bruin, Robert H.G. Kohl, Elmoubasher A.B.A. Farag, Bart L. Haagmans, Hamad Al-Romaihi, Francois Le Grange, Berend-Jan Bosch, Marion P.G. Koopmans

**Affiliations:** Erasmus Medical Center, Rotterdam, the Netherlands (C.B.E.M. Reusken, V.S. Raj, E. De Bruin, R.H.G. Kohl, B.L. Haagmans, M.P.G. Koopmans);; Al Wabra Wildlife Preservation, Doha, Qatar (C. Schilp, F. Le Grange);; Supreme Council of Health, Doha (E.A.B.A. Farag, H. Al-Romaihi);; Utrecht University Faculty of Veterinary Medicine, Utrecht, the Netherlands (B.-J. Bosch)

**Keywords:** alpaca, Vicugna pacos, Middle East respiratory syndrome coronavirus, MERS-CoV, zoonoses, camelid, dromedary, viruses, Qatar, severe acute respiratory syndrome coronavirus, SARS-CoV

**To the Editor:** Accumulating evidence indicates that dromedaries (*Camelus dromedarius*) are a reservoir for zoonotic transmission of Middle East respiratory syndrome coronavirus (MERS-CoV). Although numerous studies have looked at other livestock in the Middle East region, evidence for MERS-CoV infection has only been found in dromedaries (*1*). Extensive and continuous circulation of MERS-CoV occurs in the Al Shahaniya region of Qatar, most likely because of the presence of an international camel racing track and numerous barns holding camels (*2*,*3*). In April 2015, we investigated the MERS-CoV infection status of 15 healthy alpacas (*Vicugna pacos*) in a herd of 20 animals and 10 healthy dromedaries in a herd of 25 animals at a farm in this region (Technical Appendix). 

The herds were located at a distance of ≈200 m from each other within the barn complex and were cared for by the same animal workers, who lived in a common house between the herds at the complex. Both the alpacas and camels were kept as hobby animals. 

Serum samples were collected from all 25 animals. Nasal swabs were collected from all camels, whereas nasal, rectal, and oral swab specimens were collected only from a subset of the alpacas (Technical Appendix) because of logistical constraints. The serum samples were tested for IgG antibodies reactive with the S1 antigens of MERS-CoV and severe acute respiratory syndrome coronavirus (SARS-CoV), and titers were calculated as described previously (*4*,*5*). MERS-CoV reactivity was confirmed by using a 90% plaque-reduction neutralization test (PRNT_90_) (*3*). Swab specimens were analyzed for MERS-CoV RNA by a screening PCR targeting the upE gene (*6*). MERS-CoV–specific antibodies were detected in all alpacas and all but 1 camel by protein microarray; reciprocal titers ranged from 49 to 773 for the alpacas and were >1,280 for the camels (Figure, panel A). PRNT_90_ testing confirmed the presence of MERS-CoV–specific antibodies; reciprocal neutralizing titers ranged from 80 to 320 for the alpacas and from 80 to >2,560 for 9 camels (Figure, panel B). All swab specimens were negative by PCR (Technical Appendix). None of the serum samples were reactive to SARS-CoV S1. The microarray was also conducted for bovine CoV and human CoV-229E antigens, which were used as a proxy for the serologically closely related dromedary betacoronavirus-1 HKU23 and 229E-related camelid alphacoronaviruses, respectively (*7*). Positive binding was detected for both antigens in alpaca and dromedary (data not shown). 

**Figure:**
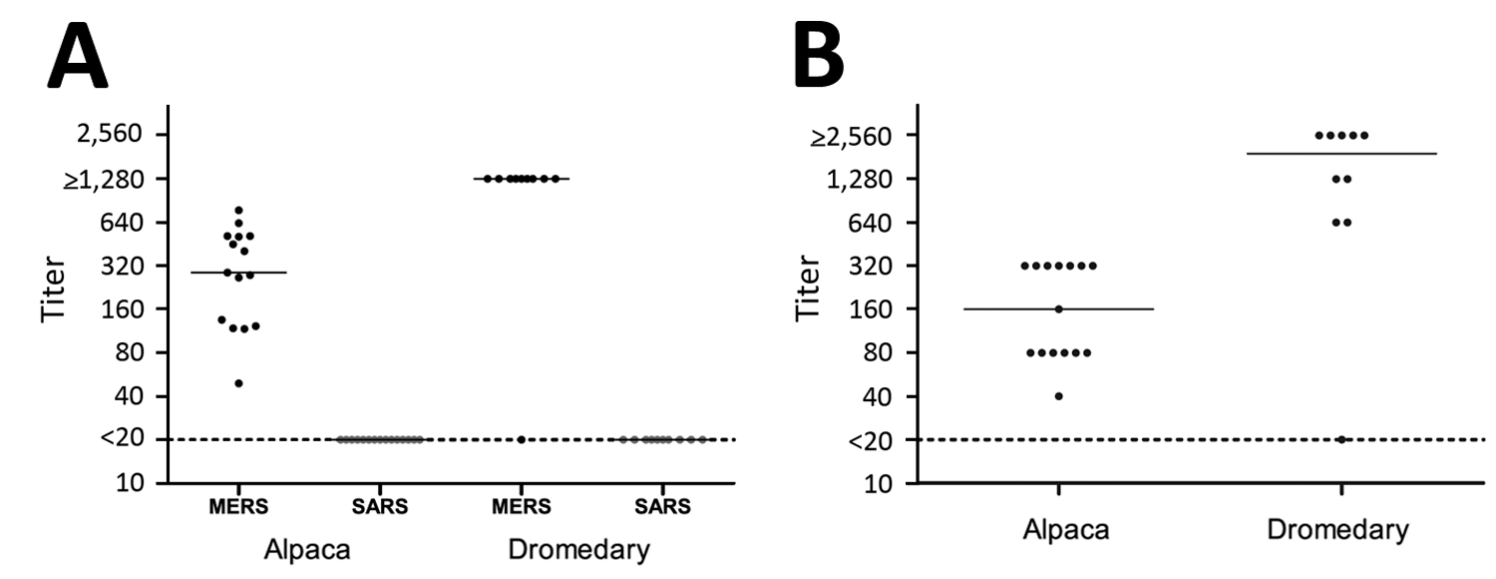
Column scatterplots of MERS-CoV reactivity of serum samples from alpaca (n = 15) and dromedaries (n = 10) in the Al Shahaniya region of Qatar, April 2015. A) Plot of alpaca and dromedary serum titers of antibodies specific for S1 antigens of 2 coronaviruses as determined by protein microarray. Titers were defined as the interpolated serum concentration that provoked a response half-way on a concentration-response curve between the minimum and maximum signal and were calculated from the inflection point of a 4-step dilution series (1:20 to 1:1,280) as described previously (*5*). B) Plot of alpaca and dromedary serum titers of MERS-CoV neutralizing antibodies as determined by PRNT_90_. The highest serum dilution neutralizing 90% of plaque formation is depicted. For both panels, solid lines indicate median, and dotted lines indicate detection limit. MERS, Middle East respiratory syndrome; CoV, coronavirus; PRNT_90_, 90% plaque-reduction neutralization test; SARS, severe acute respiratory syndrome.

Our observations prove the susceptibility of alpacas for natural MERS-CoV infection and lay the foundation for future studies to determine the potential of alpacas as another livestock reservoir for MERS-CoV. The alpacas in this study were the only alpacas in Qatar at the time and were located in a region where MERS-CoV is endemic. In a previous study, by using the same microarray technology, we found no evidence for MERS-CoV infection in alpacas from regions where MERS-CoV is not endemic (*4*). Although a study by Eckerle et al. demonstrated the potential of MERS-CoV to infect alpaca kidney cells in vitro (*8*) and alignment of mammalian DPP4 indicate that the 14 residues interacting with the MERS-CoV receptor binding domain of alpaca DPP4 are identical to that of dromedary DPP4 (Technical Appendix), the in vivo susceptibility of alpacas remained to be determined. 

The observed natural susceptibility of alpacas to MERS-CoV infection potentiates a broadening of the geographic range of MERS-CoV circulation to areas with large populations of alpacas. Alpacas are New World camelids, and the worldwide population of alpacas is estimated at 3 million animals, with ≈94% living in the high Andean regions of South America (Peru, Bolivia, Chile and Argentina), of which most are in Peru (constituting ≈88% of the world alpaca population) (http://lib.icimod.org/record/23682). Alpacas are increasingly being kept outside South America, mainly for their fleece, with estimated numbers in 2014 reaching 230,000 in the United States (http://lib.icimod.org/record/23682), 35,000 in the United Kingdom (http://www.bas-uk.com), and 150,000 in Australia (http://www.alpaca.asn.au). Although MERS-CoV has not been found in camelids other than dromedaries outside the Arabian Peninsula so far (*9*), our observations raise the question of whether other camelids could become infected if MERS-CoV were introduced to regions with large populations of alpacas and possibly other closely related camelids of the genera *Lama*, *Vicugna*, and *Camelus*.

Because the date of infection of the alpacas and camels in this study is not known, we cannot speculate on the level of susceptibility of alpacas versus dromedaries based on the observed differences in antibody titers, which were lower in alpacas. It remains to be determined whether alpacas, in parallel with dromedaries, will actually shed MERS-CoV and are capable of independent maintenance of the virus in their population. Differences in susceptibility to viral pathogens between New and Old World camelids have been observed before (*10*). Therefore, understanding the risk requires further assessment of the reservoir competence of alpacas for MERS-CoV (e.g., through experimental infections) and an assessment of MERS-CoV–related viruses present in alpacas and other camelids in different parts of the world.

**Technical Appendix.** Overview of background data and study results of alpaca and dromedary cohorts.
