# Supplementary material for: MERS-CoV Infection of Alpaca in a Region Where MERS-CoV is Endemic
Source: Emerg Infect Dis. 2016 Jun;22(6):1129–31. doi: 10.3201/eid2206.152113 (PMC4880085; doi:10.3201/eid2206.152113)
Supplement: Supplementary file 1 — Technical Appendix. Overview of background data and study results of alpaca and dromedary cohorts. [file 15-2113-Techapp-s1.pdf]

Article DOI: http://dx.doi.org/10.3201/eid2206.152113

MERS-CoV Infection of Alpaca in a Region Where MERS-CoV is Endemic

Technical Appendix

Technical Appendix Table. Overview of background data and study results of alpaca and dromedary cohorts.

				Array‡		MERS-CoV	PCR UpE§		
Animal	Age†	Sex	Origin	MERS-CoV	SARS-CoV	$PRNT_{90}$	NS	OS	AS
Alpaca									
A1	Adult	M	Qatar	287	<20	80	Neg	Neg	Neg
A2	Adult	F	Qatar	122	<20	40	ND	ND	ND
A3	Adult	M	UK	135	<20	80	Neg	Neg	Neg
A4	Adult	M	Qatar	118	<20	80	Neg	Neg	Neg
A5	Juvenile	F	Qatar	403	<20	320	ND	ND	ND
A6	Adult	F	Qatar	275	<20	80	ND	ND	ND
A7	Adult	M	UK	509	<20	320	Neg	ND	Neg
A8	Adult	M	Qatar	49	<20	80	Neg	Neg	Neg
A9	Adult	M	Qatar	265	<20	320	Neg	Neg	Neg
A10	Adult	M	Qatar	451	<20	160	Neg	Neg	Neg
A11	Adult	F	Canada	513	<20	320	ND	ND	ND
A12	Adult	F	Canada	629	<20	320	ND	ND	ND
A13	Adult	F	Canada	117	<20	80	ND	ND	ND
A14	Adult	F	Canada	513	<20	320	ND	ND	ND
A15	Adult	F	UK	773	<20	320	ND	ND	ND
Dromedary									
D1	Adult	F	Unknown	≥1280	<20	≥2560	Neg	ND	ND
D2	Adult	F	Unknown	≥1280	<20	≥2560	Neg	ND	ND
D3	Adult	F	Unknown	≥1280	<20	≥2560	Neg	ND	ND
D4	Adult	F	Unknown	≥1280	<20	≥2560	Neg	ND	ND
D5	Adult	F	Unknown	≥1280	<20	640	Neg	ND	ND
D6	Adult	F	Unknown	≥1280	<20	1280	Neg	ND	ND
D7	Adult	F	Unknown	≥1280	<20	1280	Neg	ND	ND
D8	Adult	F	Unknown	≥1280	<20	≥2560	Neg	ND	ND
D9	Adult	F	Unknown	<20	<20	<20	Neg	ND	ND
D10	Adult	M	Unknown	≥1280	<20	640	Neg	ND	ND

*MERS-CoV, Middle East respiratory syndrome coronavirus; Neg, negative result, ND, not done; NS, nasal swab, OS, oral swab, PRNT90: plaque reduction neutralization test (highest neutralizing serum dilution indicated); RS, rectal swab, RT-PCR, reverse transcription PCR; SARS-CoV, severe acute respiratory syndrome coronavirus; UK, United Kingdom.
†Juvenile = camelid age ≤2 years; adult = camelid age >2 years.
‡S1 microarray titers. Titers were defined as the interpolated serum concentration that provoked a response half-way on a concentration-response curve between the minimum and maximum signal and were calculated from the inflection point of a 4-step dilution series (1:20 to 1:1280), as previously described (*1*).
§Results RT-PCR targeting MERS-CoV UpE gene.

```
Camelus    MKTPWRVLLGLLGIAALVTLITVPAVLLSKGTDDATADSRRTYTLTDYLKNTFRLKVYTLQWVSDHEYLYRQENNILLFNAEYGNSSIFLENSTFDEFGHSINDYSVSPDRQYILFEYNY 120
Vicugna    MKTPWKVLLGLLGIAALVTVITVPVVLLNKGTDDATADSRRTYTLTDYLKNTFRLKVYTLQWVSDHEYLYRQENNILLFNAEYGNSSIFLENSTFDEFGHSINDYSVSPDRQYILFEYNY 120
           *****:*************:****.***.*******************************************************************************************

Camelus    VKQWRHSYTASYDIYDLKKRQLITEERIPNNTQWITWSPVGHKLAYVWNNDIYVKSEPNLPSQRITWTGKKDVIYNGITDWVYEEEVFSAYSALWWSPNGTFLAYAQFNDTEVPLIEYSF 240
Vicugna    VKQWRHSYTASYDIYDLKKRQLITEERIPNNTQWITWSPVGHKLAYVWNNDIYVKSEPNLPSQRITWTGEKDVIYNGITDWVYEEEVFSAYSALWWSPNGTFLAYAQFNDTEVPLIEYSF 240
           *********************************************************************:**************************************************

Camelus    YSDESLQYPKTVRIPYPKAGAVNPTVKFFVVDTSTLSPNVNATSRQIVPPASVLIGDHYLCGVTWVTEKRISLQWIRRIQNYSIMDVCDYDESTGRWASSVGRQHIETSTTGWVGRFRPA 360
Vicugna    YSDESLQYPKTVRIPYPKAGAVNPTVKFFVVDISTLSPNVNATSRQIVPPASVLIGDHYLCGVTWVTEKRISLQWIRRIQNYSIMDVCDYDESTGRWASSVGRQHIETSTTGWVGRFRPA 360
           ******************************** ***************************************************************************************

Camelus    EPHFTSDGSSFYKIISNEEGYKHICHFQTDKRNCTFITKGAWEVIGIEALTRDYLYYISNEHKGMPGGRNLYKVQLNDYTKVTCLTCELDPERCQYYSASFSKEAKYYQLRCSGPGLPLY 480
Vicugna    EPHFTSDGSSFYKIISNEEGYKHICHFQTDKRNCTFVTKGAWEVIGIEALTSDYLYYISNEHKGMPGGRNLYKVQLNDYTKVTCLTCELDPERCQYYSVSFSKEAKYYQLRCSGPGLPLY 480
           ************************************:************** **********************************************.*********************

Camelus    TLHSSSSDKELRVLENNSALENMLQEVQMPTKKLDFINMHETKFWYQMILPPHFDKSKKYPLLIDVYAGPCSQKADTIFRLNWATYLASTENIIVASFDGRGSGYQGDKIMHAINRRLGT 600
Vicugna    TLHSSSSDKELRVLENNSALENMLQEVQMPTKKLDFINMHETKFWYQMILPPHFDKSKKYPLLIDVYAGPCSQKADTIFRLNWATYLASTENIIVASFDGRGSGYQGDKIMHAINRRLGT 600
           ************************************************************************************************************************

Camelus    FEVEDQIEATRQFSKMGFVDDKRIAIWGWSYGGYVTSMVLGAGSGVFKCGIAVAPVSKWEYYDSVYTERYMGLPTPQDNLDYYRNSTVMSRAENFKQVEYLLIHGTADDNVHFQQSAQIS 720
Vicugna    FEVEDQIEATRQFSKMGFVDDERIAIWGWSYGGYVTSMVLGAGSGVFKCGIAVAPVSKWEYYDSVYTERYMGLPTPQDNLDYYRNSTVMSRAENFKQVEYLLIHGTADDNVHFQQSAQIS 720
           *********************:**************************************************************************************************

Camelus    KALVDAGVDFQTMWYTDEDHGIASSTAHQHIYTHMSHFLKQCFSLP 766
Vicugna    KALVDAGVDFQTMWYTDEDHGIASSTAHQHIYTHMSHFLKQCFSLP 766
           **********************************************
```

Technical Appendix Figure. Alignment of DPP4 protein sequences of dromedary camel (*Camelus dromedaries;* seq.ID: AIG55259.1) and alpaca (*Vicugna pacos;* seq.ID: XP_006196278.1). Nonidentical sequences indicated in blue. MERS-CoV S1 interacting residues indicated in red.

Reference

1. Koopmans M, de Bruin E, Godeke GJ, Friesema I, van Gageldonk R, Schipper M, et al. Profiling of humoral immune responses to influenza viruses by using protein microarray. Clin Microbiol Infect. 2012;18:797–807. PubMed http://dx.doi.org/10.1111/j.1469-0691.2011.03701.x
